# Long-distance association of topological boundaries through nuclear condensates

**DOI:** 10.1073/pnas.2206216119

**Published:** 2022-08-01

**Authors:** Amir Gamliel, Dario Meluzzi, Soohwan Oh, Nan Jiang, Eugin Destici, Michael G. Rosenfeld, Sreejith J. Nair

**Affiliations:** ^a^Department of Medicine, University of California San Diego, La Jolla, CA 92093;; ^b^HHMI, University of California San Diego, La Jolla, CA 92093;; ^c^Department of Neurosciences, University of California San Diego, La Jolla, CA 92093

**Keywords:** chromosome architecture, transcription, condensate biology

## Abstract

The eukaryotic genome is partitioned into distinct topological domains separated by boundary elements. Emerging data support the concept that several well-established nuclear compartments are ribonucleoprotein condensates assembled through the physical process of phase separation. Here, based on our demonstration that chemical disruption of nuclear condensate assembly weakens the insulation properties of a specific subset (∼20%) of topologically associated domain (TAD) boundaries, we report that the disrupted boundaries are characterized by a high level of transcription and striking spatial clustering. These topological boundary regions tend to be spatially associated, even interchromosomally, segregate with nuclear speckles, and harbor a specific subset of “housekeeping” genes widely expressed in diverse cell types. These observations reveal a previously unappreciated mode of genome organization mediated by conserved boundary elements harboring highly and widely expressed transcription units and associated transcriptional condensates.

## Significance

The cellular mechanisms organizing chromosomal architecture in the eukaryotic genome are not yet well understood. Here, based on our demonstration that chemical disruption of nuclear condensate assembly weakens the insulation properties of a specific subset (∼20%) of topologically associated domain (TAD) boundaries, we were able to reveal that these boundary elements are characterized by high transcriptional activity, striking spatial clustering, and an augmented presence of long-distance/interchromosomal interactions between transcription units widely expressed in diverse cell types (i.e., boundary-associated “housekeeping” genes), thus providing insights into the physical basis for TAD-boundary establishment. Therefore, these results support a model whereby transcription contributes to chromatin compartmentalization and hierarchical genome organization, potentially through the association of transcriptional condensates with membraneless nuclear organelles.

Microscopic studies and sequencing-based approaches have revealed that the eukaryotic genome is hierarchically organized and partitioned into distinct compartments (1, 2). For instance, the banding pattern of the chromosomes correlates with euchromatin and heterochromatin regions (3, 4), and the mammalian chromosomes occupy distinct positions within the nucleus, referred to as chromosomal territories (5). Principal component analysis of the Hi-C data revealed the partitioning of the genome into A and B compartments (6, 7), with A compartments tending to be gene rich, transcriptionally active, and exhibiting the epigenetic features of active chromatin. In contrast, B compartments contained gene-poor genomic regions that are transcriptionally less active and associated with repressive nuclear structures such as the nucleolus and lamin-associated domains (LADs) (8). Analysis of contact maps from high-resolution Hi-C data sets revealed the presence of self-associating chromosomal domains known as topologically associated domains (TADs) (9, 10), providing an insight into chromosomal topography. Genomic regions within the TADs were found to engage in a high frequency of interactions, separated from adjacent TADs by boundary elements. While the genomic compartments such as A/B and TADs were defined primarily through bioinformatic analysis of data derived from conformation capture assays, the presence of self-associating domains has been further confirmed by microscopic studies (11). The cellular mechanisms that drive genomic compartmentalization and the establishment of boundaries remain incompletely understood.

The emergence of the biophysical process of liquid–liquid phase separation (LLPS) as a potential driving force in forming several membraneless cellular organelles (12, 13) raises the question of whether investigating this process might provide additional insights into TAD-boundary organization. Indeed, the eukaryotic nucleus harbors several prototypic membraneless organelles, some of which are formed by the process of LLPS (13–15). The phase separation property of the repressive histone mark H3K9me3-binding protein, HP1α, is critical in forming heterochromatin domains in the nucleus (16, 17). Similarly, the chromatin-binding protein, BRD4, effectively induces phase separation of H3K27ac histones associated with actively transcribed genomic regions (18). Further, condensate formation of RNA Pol II and several transcription factors and cofactors are critical for gene regulation mediated by enhancers (19–24). Together, these findings are consistent with the hypothesis that the immiscible condensates formed at transcriptional sites, heterochromatic and euchromatin domains in the eukaryotic chromatin, together contribute to spatial segregation of the genome into distinct domains based on transcriptional activity.

However, technically, it remains a challenge to determine whether the LLPS-based mechanisms contribute to the formation of the chromatin organizing units such as TADs and A/B compartments in the intact cells. Because of a clear overlap of transcriptionally less active heterochromatin with the B compartments (8) and transcriptionally active genomic regions with the A compartments (25), it is tempting to hypothesize that genomic compartmentalization is partly driven by immiscible condensates assembled at these domains.

Treatment of cells with the aliphatic alcohol 1,6-hexanediol (1,6-HD) has been shown to disrupt many cellular membraneless organelles by weakening the hydrophobic interactions between molecules (26, 27). This provides a valuable tool for determining the regulatory roles of condensates in various biological processes (21, 26). Our data reveal that, in human embryonic stem cells, the insulation property of ∼20% of TAD boundaries was significantly diminished within 5 min of treatment with 1,6-HD. This phenotype was largely rescued by drug wash away, supporting the hypothesis that the loss of these boundaries reflected the transient disruption of membraneless structures assembled at the boundaries. In accordance with the observation that high transcription levels characterize the 1,6-HD sensitive boundaries, chemical inhibition of transcription weakened the insulation of the same cohort of boundary regions. Hi-C and immuno-DNA fluorescence in situ hybridization (FISH) experiments revealed that these responsive boundaries, harboring highly expressed transcription units, were clustered and spatially proximal to each other. Together these results indicate that a subset of boundary elements is maintained by transcription-induced membraneless compartments, which contribute to a previously unappreciated type of genome organization in a cell type–independent manner.

## Results

### Effect of Aliphatic Alcohol on Chromatin Organization.

**Fig. 1.**
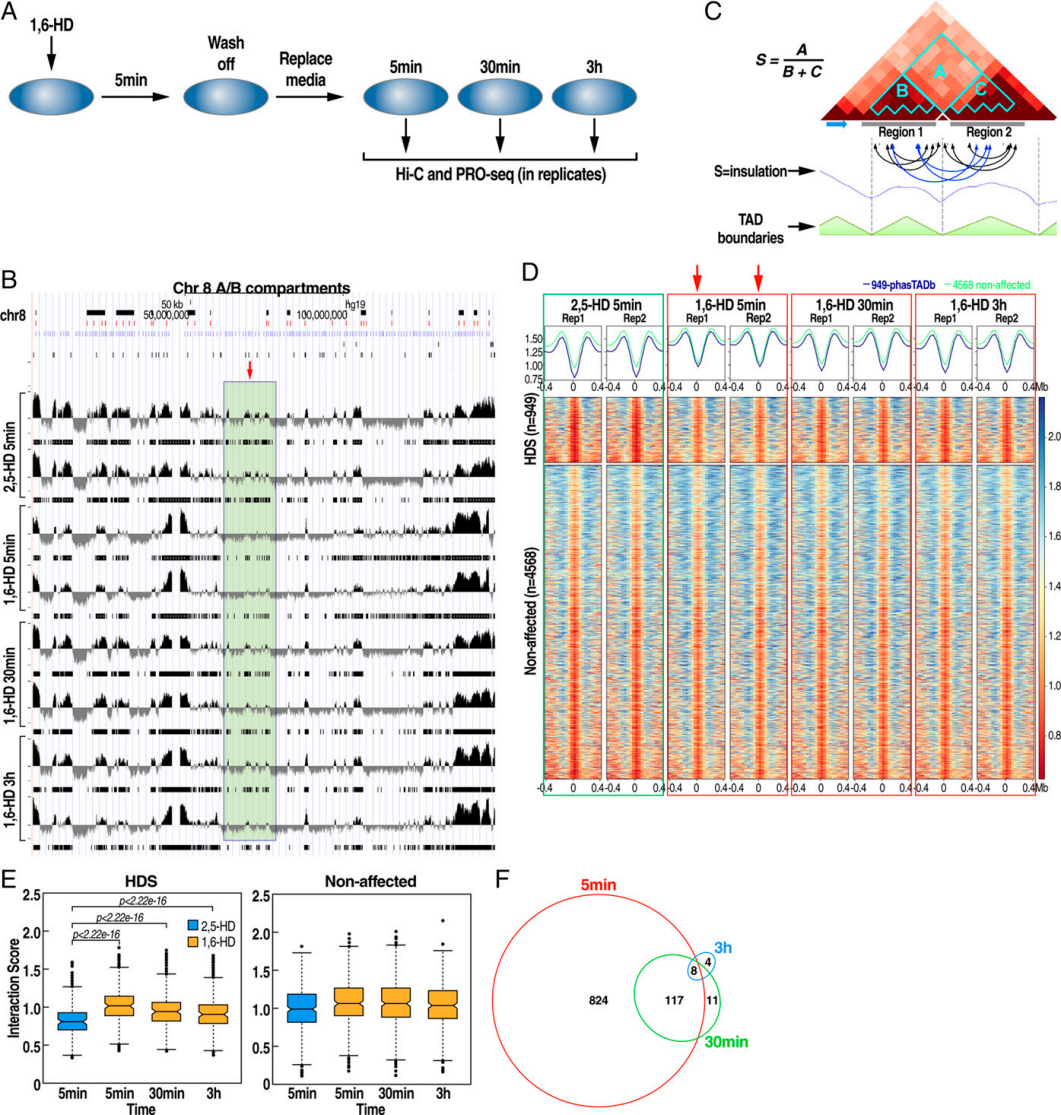
The aliphatic alcohol 1,6-HD induces a transient reduction in insulation for a subset of TAD boundaries. (*A*) Schematics of the experimental strategy. (*B*) Browser example from chromosome 8 showing PC1 principal component–derived compartments (A, positive values; B, negative values) with a region showing small changes to the A/B compartment only after a 5-min treatment. (*C*) Schematic representation of the insulation score calculation. Insulation score minima were used to define TAD boundaries. (*D*) Heat map plotting all insulation scores across all boundaries in the specified time points. A total of 949 significantly affected (HDS) boundaries (*Top*) were identified after a 5-min treatment in replicates (red arrows) vs. 4,568 unaffected boundaries (*Bottom*). (*E*) Quantitation of the interaction score shown in *D*. (*F*) Venn diagram of significantly affected boundaries at each time point showing most boundaries affected after 5 min rapidly recover over time.

The aliphatic alcohol 1,6-HD has been used extensively to test the material property and the contribution of RNA–protein condensates in the assembly of membraneless compartments in vitro and in vivo (26–29). The aliphatic alcohol 1,6-HD is suggested to weaken the protein–protein and protein–RNA hydrophobic interactions resulting in the dissociation of membraneless condensates. To examine the contribution of such putative phase-separated, 1,6-HD–sensitive assemblies on chromatin architecture, we treated human embryonic stem cells (RUES1) with 7% 1,6-hexanediol for 5 min. The chemical was washed away after 5 min and replaced with fresh media. The cells were collected for in situ Hi-C and precision nuclear run-on sequencing (PRO-seq) experiments at 5 min, 30 min, and 3 h after wash-off (Fig. 1*A*). In all these experiments, aliphatic alcohol 2,5-hexanediol (2,5-HD), a related compound with no apparent impact on membraneless organelle assembly and transcription (21, 27), was used to treat the control group.

In situ Hi-C library sequencing from two biological replicates for each time point generated ∼200 million reads, of which 160 million uniquely aligned to the human reference genome (*SI Appendix*, Dataset S1). We constructed contact count matrices using genomic bins of sizes 40 kb to identify TAD boundaries, 500 kb to generate A/B compartment profiles, and heat maps representing low-resolution intra and interchromosomal contacts. Correlation coefficient calculations exhibited high similarity between replicates (*SI Appendix*, Fig. S3 *B* and *C*). Therefore, the replicates were pooled for further analysis when needed. When we compared A/B compartment profiles in the different treatments, we found only rare changes in compartments, namely 0, 15, and 3 genomic bins for the 5 min, 30 min, and 3 h 1,6-HD–treated samples (false discovery rate [FDR] <0.05), respectively, out of 5,726 total bins when compared to the 2,5-HD control (Fig. 1*B*), suggesting the absence of any global changes in genome architecture. Next, to identify TAD boundaries, we calculated insulation scores between adjacent genomic regions to examine the impact of the 1,6-HD on TAD organization (Fig. 1*C*). We leveraged the insulation scores derived from two independent experiments to identify the boundaries exhibiting significantly altered insulation properties using a generalized linear model (GLM) (*SI Appendix*, Dataset S2). This analysis revealed a significant change in the insulation score in ∼20% of boundaries in RUES1 cells (949/5,517) at the 5-min time point compared to 2,5-HD–treated samples (Fig. 1*D* and *SI Appendix*, Fig. S1 *A–C*). However, these boundaries gradually returned to normal insulation level by 3 h (Fig. 1 *D* and *E*). In comparison, 1,6-HD did not affect the insulation scores of the remaining 4,568 boundary elements (Fig. 1*D*). We refer to these as 1,6-hexanediol–sensitive TAD boundaries (hereafter HDS boundaries). The overlap between affected boundaries at different time points further illustrates that most of the boundaries are affected within 5 min of 1,6-HD treatment. After removing 1,6-HD, these boundaries recovered their insulation property (Fig. 1 *D*–*F*).

### Features of HDS Boundaries.

**Fig. 2.**
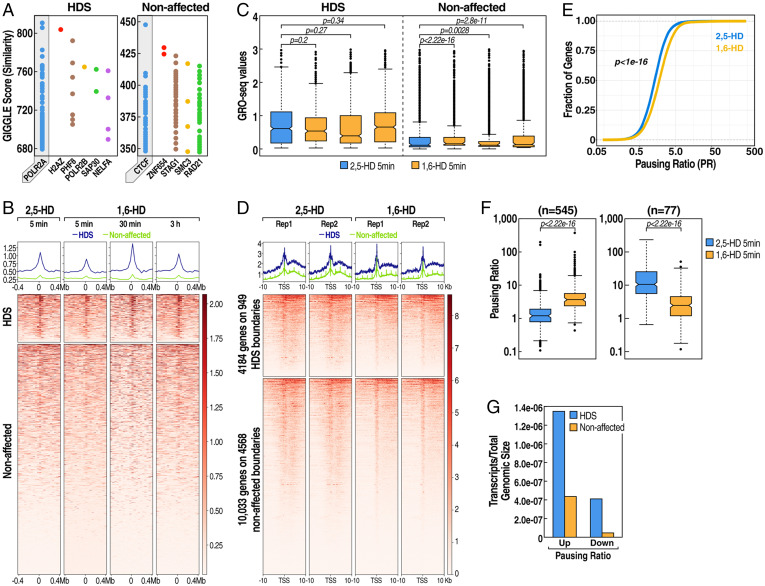
HDS boundaries are characterized by high transcription levels and undergo transcriptional pausing after 1,6-HD treatment. (*A*) Enrichment of transcription factor ChIP-seq datasets on HDS and unaffected boundaries from the Cistrome database. Each dot on the resulting plot represents a ChIP-seq sample with its corresponding GIGGLE score, where higher GIGGLE scores indicate more enrichment. (*B*) Heat map showing PRO-seq transcription levels on HDS (*Top*) or unaffected (*Bottom*) centered on boundaries at each time point. (*C*) Quantitation of PRO-seq transcription levels as shown in *B*. (*D*) Heat maps of PRO-seq transcription at HDS or unaffected boundaries after a 5-min 1,6-hexanediol treatment in replicates, centering on gene transcription start sites (TSSs) for genes within the given regions. (*E*) Cumulative pausing ratio plots of all genes in control (2,5-HD) (blue) or 1,6-HD–treated (yellow) RUES1 cells. (*F*) Pausing ratios of significantly more paused (*Left*, *n* = 545) or less paused (*Right*, *n* = 77) genes in control (2,5-HD) or 1,6-HD 5-min–treated RUES cells. (*G*) Relative enrichment of 1,6-HD deregulated genes with increased (up) or decreased (down) pausing overlapping HDS vs. unaffected boundaries. Transcript numbers were normalized to the total genomic size of HDS and unaffected regions.

To identify the genomic features that distinguish the HDS and non-HDS boundaries, we analyzed the published and annotated chromatin immunoprecipitation–sequencing (ChIP-seq) database (cistrome.org/) of human transcription factors and regulators to search for known factors that are most enriched in each of the two classes of boundaries (Fig. 2*A*). The most enriched components on the non-HDS boundaries were the architectural proteins CTCF and cohesin subunits, which are known to be a common feature of the eukaryotic TAD-boundary regions (9, 30, 31). ChIP-seq for CTCF and cohesin subunit RAD21 showed that the recruitment of these proteins to HDS and nonaffected boundaries were equivalent in both categories and were largely unaffected by 1,6-HD treatment (*SI Appendix*, Fig. S2*A*). This suggests that the recruitment of the architectural proteins CTCF and cohesin is equivalent on both boundary categories and is not the critical underlying feature of differential sensitivity to 1,6-HD. In contrast, cistrome analysis revealed that HDS boundaries were enriched in regulatory proteins such as RNA Polymerase II and other transcription regulators (Fig. 2*A*), suggesting that HDS boundaries may be highly transcribed.

### HDS Boundaries Are Transcriptionally Highly Active.

To directly test the transcriptional activity of the TAD boundaries, we performed global run-on sequencing (GRO-seq) (32) or PRO-seq (33) on nuclei collected from RUES1 cells with equivalent results. As opposed to the steady-state transcript information obtained from RNA-seq, PRO-seq reveals real-time engagement of RNA Pol II on the chromatin by directly measuring the nascent RNA synthesis (32, 33). RUES1 cells were treated with 2,5-HD or 1,6-HD, followed by a recovery period of 5 min, 30 min, and 3 h. Heat map of PRO-seq data centered on boundaries, revealed enrichment of transcription consistent with previous descriptions (9). The transcription at HDS boundaries was significantly higher than that from the unaffected boundaries (Fig. 2 *B* and *C*), consistent with the cistrome data indicating a higher level of transcription regulators in the HDS boundaries (Fig. 2*A*). Treatment with 1,6-HD appeared to significantly attenuate transcription of nascent RNAs from HDS-boundary regions 5 min postexposure (Fig. 2 *B* and *C*). The nascent RNA transcription returned to the level of the control treatment condition 3 h after the treatment, thus paralleling the recovery pattern of the chromatin architecture at the same time points (Fig. 1 *D* and *E*). Together these data suggest that a high transcription level is a crucial feature of HDS boundaries.

Since the most substantial effect of 1,6-HD on Hi-C architecture was at 5 min, we focused our efforts at that time point to better understand the transcriptional changes occurring during that time interval. We, therefore, performed additional PRO-seq assays at the 5-min time point on RUES1 cells treated with either 2,5-HD or 1,6-HD in replicates (*SI Appendix*, Fig. S2 *C* and *D*). Analysis targeting de novo transcripts revealed that 1,6-HD treatment led to 1,166 down-regulated genes and 520 up-regulated genes, suggesting dysregulation of transcription, with a preponderance of down-regulated genes (*SI Appendix*, Fig. S2 *E–G*). Next, we examined the PRO-seq profiles of all genes that overlap either with HDS or nonaffected boundaries (Fig. 2*D*). This approach revealed several interesting features. Although the HDS boundaries consisted of only 19.58% of TAD boundaries, 38.4% of the entire boundary-derived transcripts were from HDS-boundary regions. This suggests a mechanism underlying the chromatin organization involving active transcription units at the HDS-boundary regions. Second, the heat map of PRO-seq tags over the 10-kb window around transcription start sites revealed that 1,6-HD treatment resulted in a subtle but distinct transcript profile consistent with promoter-proximal pausing of RNA Polymerase II, characterized by reduced tag density over gene bodies, with an increased density at the transcription start sites (Fig. 2*D*). Indeed, calculation of the pausing index (34) of all genes suggested a small but statistically significant trend for widespread transcriptional pausing following 1,6-HD treatment (Fig. 2*E*), with 545 genes exhibiting significantly increased pausing and only 77 showing reduced pausing (Fig. 2*F*). While our PRO-seq data suggest a widespread trend for transcriptional pausing across many genes, transcripts on HDS boundaries were disproportionally more affected than those on non-HDS boundaries (Fig. 2*G*). Taken together, our results reveal that HDS boundaries exhibit much higher levels of transcription than non-HDS boundaries, and thus even a global effect of 1,6-HD on transcription predominantly affects HDS boundaries. Further, the transcriptional pausing is transient and released after a 3-h recovery following the chemical wash-away, suggesting that the treatment predominantly affects HDS-boundary regions rather than causing the collapse of transcriptional machinery across the genome. This result further illustrates that active transcription, rather than the accumulation of transcription initiation apparatus (as in paused gene promoters), represents the underlying mechanism for the proper maintenance of HDS boundaries.

### HDS Boundaries Are Sensitive to Transcriptional Inhibition.

**Fig. 3.**
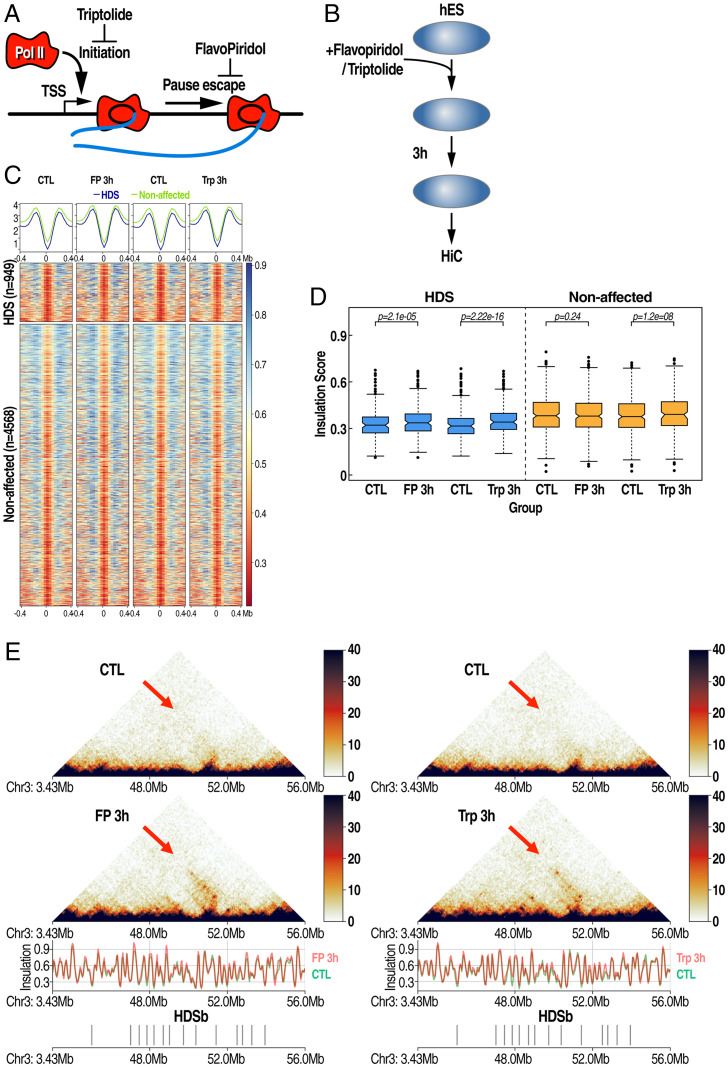
Transcription inhibitors recapitulate insulation reduction on HDS boundaries. (*A*) Schematic of the mode of action of an inhibitor of transcriptional initiation (triptolide) or transcriptional elongation (flavopiridol). (*B*) Schematic of the treatment strategy using inhibitors for downstream Hi-C. (*C*) Heat map plotting insulation scores on significantly affected (HDS, *Top*) vs. unaffected boundaries (*Bottom*) in flavopiridol- and triptolide-treated cells. (*D*) Quantitation of interaction scores shown in *C*. (*E*) Plots of Hi-C matrices in control, flavopiridol, or triptolide RUES cells treated with inhibitors for 3 h. Arrows highlight increased long-range interactions after treatment over regions with HDS-boundary (HDSb) clusters.

Although the above data reveal a correlation between changes in transcription and HDS-boundary strength, it is possible that 1,6-HD may alter the nuclear architecture, leading to alterations in transcription, or vice versa. To directly determine the relationship between active transcription and the maintenance of HDS boundaries, we treated RUES1 cells with either the drug triptolide, which inhibits transcription initiation, or flavopiridol, which inhibits transcription elongation by blocking the CDK9 kinase activity and thereby reducing the elongating form of RNA Polymerase II (Fig. 3*A*) (35). RUES1 cells were treated with the transcription inhibitors for 15 min and 1 and 3 h, after which cells were collected for Hi-C experiments. This treatment effectively inhibited the transcription of genes by 3 h, based on the level of two highly expressed nascent transcripts of the genes BAMBI and FOS (*SI Appendix*, Fig. S3*A*). RUES1 cells were treated with flavopiridol and triptolide, and the samples were cross-linked and used for Hi-C library preparation (Fig. 3*B*). This experiment revealed that transcriptional inhibition resulted in a modest but consistent reduction in the insulation score, specifically at HDS boundaries but not on the unaffected boundaries (Fig. 3 *C* and *D*). The weakening of the boundary resulted in increased inter-TAD interactions between TADs flanking the HDS boundaries (Fig. 3*E*). These data further support the hypothesis that transcriptional activities in these genomic regions facilitate the TAD-boundary formation.

### Genomic and Spatial Clustering of HDS Boundaries.

**Fig. 4.**
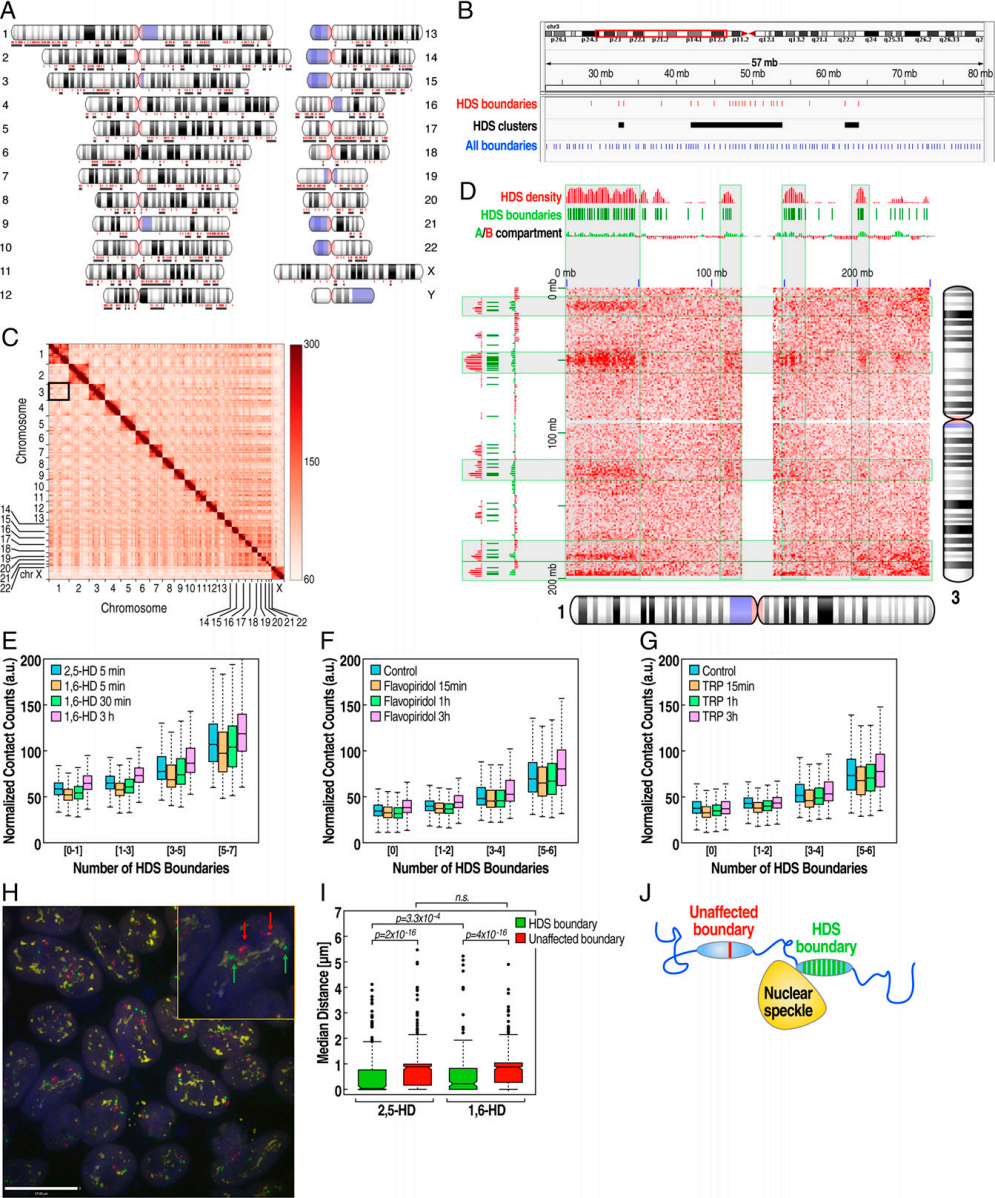
HDS boundaries are organized in clusters that exhibit enhanced interchromosomal interactions and increased association with interchromatin granule clusters (speckles). (*A*) Whole-genome chromosome schematic shows HDS boundaries (red dots) and clusters (black lines). (*B*) Example of HDS boundary clusters on chromosome 3. HDS boundaries (red lines), HDS clusters (black bars), and all boundaries (blue lines). (*C*) Hi-C interaction matrix showing all chromosomes. The black box highlights the location of interchromosomal interactions between chromosomes 1 and 3. (*D*) Example of Hi-C interaction between chromosomes 1 and 3. Increased interactions (red) are enriched over HDS boundaries (green dashes) and HDS-boundary density (red density plot). (*E–G*) Normalized interchromosomal contact counts between 4-Mb genomic regions grouped by the number of HDS boundaries per region in the different 1,6-HD–treated time points (*E*), flavopiridol (*F*), and triptolide (*G*) treatments. (*H*) Example of association of nuclear speckles with HDS-boundary cluster vs. unaffected boundary on the same chromosome by immune-DNA FISH. Representative immune-DNA FISH image showing cells stained with the nuclear speckle marker SON (yellow), hybridized with probes targeting an unaffected boundary (red) and an HDS-boundary cluster (green). *Inset* shows a higher magnification of one cell, showing two alleles of an unaffected (red arrow) and an HDS-boundary cluster (green arrow). (*I*) Quantitation of immune-DNA FISH images shows that HDS boundary clusters (green) are significantly closer to the nuclear speckle marker (SON) than unaffected boundaries (red). The 1,6-HD treatment increases the median distance between HDS boundary and nuclear speckles, but it has no impact on the non-HDS–boundary association with speckles (n.s. - not significant). (*J*) Visual interpretation of immune-DNA FISH data.

The eukaryotic genome is hierarchically organized into distinct compartments based on the transcriptional activity (2, 7). Phase-separated nuclear condensates have been proposed as a potential organizing platform for three-dimensional (3D) chromatin architecture through a surface tension–driven coalescence (36). This led us to hypothesize that transcriptionally active HDS boundaries might associate spatially to add another layer of architectural complexity to the genome. Examination of genome-wide distribution of HDS boundaries revealed that these boundary regions tended to occur in clusters in the genome (Fig. 4*A*). The HDS boundaries were distributed in all chromosomes with an average of 4 HDS boundaries/cluster (minimum 2, maximum 29 boundaries/cluster) (Fig. 4*B*), with 86% occurring in clusters. The rest of the boundary elements were uniformly distributed across the chromosomes.

For further analysis, we filtered out boundaries that were 1 Mb proximal to centromere regions due to poor mappability. We generated contact matrices for all chromosomes to detect intrachromosomal and interchromosomal interactions using 4-Mb bins (Fig. 4*C*). This revealed that total interactions are dominated by intrachromosomal interactions, as previously described. Interestingly, we also observed specific patterns in several interchromosomal interactions that have not previously been characterized. Most of the interaction domains between chromosomes were large genomic regions (on average 5 to 10 Mb), overlapping clustered HDS boundaries (Fig. 4*D*).

To systematically assess the transchromosomal association of clustered HDS boundaries, we calculated the number of HDS boundaries per 4-Mb bin and examined their interactions. First, we observed that bins containing a relatively high number of HDS boundaries had increased interchromosomal interactions (Fig. 4 *E* and *F*). This was not due to differences in the total number of boundaries in each bin (*SI Appendix*, Fig. S4*A*). Second, after 5 min of 1,6-HD treatment, interactions were reduced but subsequently recovered by 3 h after treatment (Fig. 4 *E* and *F*). Moreover, inhibition of transcription using flavopiridol or triptolide also reduced interchromosomal interactions rapidly within 15 min of treatment and then slowly recovered, matching the results obtained by 1,6-HD treatment (Fig. 4 *F* and *G*). These results suggested that highly transcribed, clustered HDS boundaries engaged in transchromosomal interaction with other HDS boundaries. Our analysis, therefore, reveals a previously unappreciated interchromosomal network of interacting, highly transcribed TAD-boundary elements.

Actively transcribed genomic regions have been associated with nuclear speckles (37, 38). Recent high throughput studies have further revealed that functional genomic compartments (A compartments) are highly associated with nuclear speckles. In contrast, the transcriptionally inactive compartments (B compartments) are associated with nucleolus- or lamin-associated domains (39, 40). Based on these findings, we hypothesized that the spatial proximity observed between HDS boundaries might be mediated through their association with membraneless nuclear compartments, such as nuclear speckles. To test this hypothesis, we performed immuno-DNA FISH using DNA probes targeting the HDS and non-HDS boundary located on chromosome 3 along with the nuclear speckles marker protein, SON. HDS boundaries were significantly closer to nuclear speckles than unaffected boundaries (Fig. 4 *H* and *I*). We next examined the impact of 1,6-HD on these interactions. DNA-FISH analysis revealed that the spatial proximity between HDS boundaries was significantly reduced with 1,6-HD treatment, whereas no significant difference in spatial distance was observed between non-HDS boundaries (Fig. 4*I*). This finding was confirmed with a second pair of DNA-FISH probes targeting HDS and non-HDS boundaries on chromosome 7 (*SI Appendix*, Fig. S4*E*). This suggests that highly expressed HDS boundaries utilize a mechanism of spatial chromatin organization using membraneless nuclear organelles as anchor points. Finally, to gain insight into the functional basis of this HDS-boundary organization, we examined 54 human tissue gene expression datasets from the Genotype-Tissue Expression (GTEx) database. We selected the top 1% of highly expressed genes common to all cell types (*SI Appendix*, Fig. S4*B*). When taking into account that HDS boundary clusters account for ∼20% of the genome (*SI Appendix*, Fig. S4*C*), we found that the top 1% of the highly expressed genes common to all cell types are fivefold more enriched on HDS-boundary clusters in comparison to the rest of the genome (*SI Appendix*, Fig. S4*D*). These data suggest that highly transcribed HDS-boundary clusters are organized to ensure robust expression of a subset of essential genes, often referred to as “housekeeping genes,” required in most cell types.

## Discussion

Our study provides insights into the physical mechanisms underlying the establishment of a subset of TAD-boundary regions. In this manuscript, we show that ∼20% of TAD-boundary elements, characterized by robust transcriptional activity and the recruitment of transcriptional apparatus, are sensitive to the disruption of hydrophobic interactions by the aliphatic alcohol 1,6-hexanediol. Because of the disruptive effects of 1,6-HD on a wide variety of biomolecular condensates (26, 27), these data support a regulatory role for ribonucleoprotein condensates, potentially with LLPS-like properties, at this subset of boundaries. We propose that the physical forces derived from the interaction between transcriptional condensates on these boundaries and subnuclear organelles, particularly the nuclear speckles, contribute to a previously unappreciated cellular mechanism underlying the three-dimensional organization of the genome.

The underlying mechanisms of chromosomal partitioning are not entirely understood. Studies have explored the enrichment of various chromatin-binding proteins and epigenomic markers to explain the organization of TADs. In mammalian cells, CTCF and cohesin subunits are highly enriched in a large proportion of boundary regions; however, CTCF and cohesin themselves are not sufficient to demarcate the boundary elements (9). Overrepresentation of the highly transcribed housekeeping genes has been a key feature of the TAD boundaries (9, 41). In accord with these observations, we found that 19.5% of boundary regions in human embryonic stem cells are highly transcribed. The insulation of these regions is significantly reduced upon the inhibition of active transcription. This resulted in increased inter-TAD genomic interactions at a rate that correlates with the insulation score reduction after 1,6-HD treatment. Notably, the TAD boundaries that were transiently affected by 1,6-HD were similarly affected by transcriptional inhibition using two different transcriptional inhibitors. One caveat to our findings is that some of the changes observed in boundary insulation and transcription following treatment with 1,6-HD are of small magnitude. However, the fact that they were highly reproducible in the replicates allowed us to identify these changes as robustly significant.

Moreover, the fact that we could essentially reproduce the effect on boundary insulation with two drugs inhibiting transcription (flavopiridol and triptolide), which have very different mechanisms of action, suggests this finding is consistent and robust, despite the relatively small effect size. Our data, therefore, support the model that highly active transcription units maintain a subset of boundary regions. We propose that the transcriptional condensates assembled at high concentrations in these regions are required to maintain the boundary function. A study of early mouse development reported a lack of impact of transcription-inhibiting drugs on the chromatin architecture (42), perhaps reflecting the difference in the transcriptional inhibitor used in that experiment. In support of this explanation, another study that used flavopiridol, the same drug used in our study, observed similar drug effects on actively transcribed boundary elements in flies (43).

An equally intriguing observation is the resistance of the majority of TAD boundaries to 1,6-HD. It is possible that the condensates that organize these boundaries are not sensitive to 1,6-HD, as several types of molecular forces that drive the assembly of biomolecular condensates have differential sensitivities to various chemicals (44). It is also likely that the organization of 1,6-HD insensitive boundaries is transcription independent, and the underlying molecular interactions are nonhydrophobic in nature. These TAD domains, potentially organized by the loop extrusion mechanism mediated by the motor function of the cohesin ring and the insulator function of CTCF (45–47), apparently do not rely on condensate assembly. However, it remains unclear why transcription at HDS boundaries increases their insulation properties. One possible mechanism is that the transcriptional machinery involving the recruitment of Pol II and the unwinding of the DNA strand creates a structural impediment that slows the progression of the loop extrusion machinery, thereby increasing the likelihood of more stable loops and increased insulation between TADs.

Transcription has also been suggested to be responsible for short-range loop interactions, detected as “stripes,” that correlate to enhancer–promoter and promoter–promoter interactions even in the absence of CTCF or cohesin binding (48, 49). Such short-range interactions could thus potentially hinder longer-range interactions across HDS boundaries when transcription occurs at high levels. Alternatively, the locally transcribed mRNA itself may regulate the condensate formation and dynamics during transcription (50) that could serve as a barrier to interactions across HDS boundaries. Finally, it has been recently shown that CTCF has a high affinity for RNA and that a subset of CTCF binding events on chromatin can be regulated by RNA interaction (51). Therefore, it can be hypothesized that nascent RNA transcribed at HDS boundaries may potentially interact locally with bound CTCF to promote loop stabilization, even while not affecting the CTCF occupancy per se. Further work will be required to distinguish between these possibilities.

Many reported long-distance genomic interactions span multiple TADs and even different chromosomes (52–58). The precise mechanisms of such long-distance genomic interactions are not well understood. Recent studies have suggested a role for subnuclear structures such as nuclear speckles and nucleoli in the spatial segregation of the genome (39, 40). Nuclear speckles have also been suggested to anchor long-distance genomic interactions (21, 37, 38). Our Hi-C data indicate that transcriptionally active HDS boundaries are positioned in spatial proximity. Immuno-DNA FISH supports that these regions have an increased propensity to be in the proximity of nuclear speckles. These associations observed in the Hi-C matrix are conserved across multiple cell types and embryonic stem cells, indicating that this organization may be established early in development. These results align with previous concepts such as “transcription hubs/factories” (59, 60) that suggest multiple transcribed regions from different chromosomes may interact at “hubs” to drive efficient transcription (61). Indeed, other Hi-C–based studies have reported increased global interactions between highly transcribed regions (62, 63). Together, these studies, in concert with the data presented in this paper, provide a mechanism for organizing a 3D genome using conserved architectural elements and coopting subnuclear architectural structures.

In contrast to the mounting evidence for the spatial segregation of chromatin as a result of chromatin-activity states and phase separation (16–18, 64), in our study, 1,6-HD at 7% concentration failed to cause any significant disruption of the A/B-compartment organization. Although a modest but irreversible change in A/B compartments after 1,6-HD treatment has been observed in HeLa cells (65), we did not see any significant changes in A/B-compartment organization following 1,6-HD treatment in RUES1 cells. The gained interactions we observed between TADs were fully recovered within 3 h of wash-off. Our data thus suggest that higher-order chromatin organizations, such as A/B compartmentalization, are not sensitive to 1,6-HD, which is widely used to disrupt the weak multivalent protein–protein and protein–RNA interactions that are hydrophobic in nature (28, 29). In contrast, 1,6-hexanediol produced significant but incomplete dispersal of heterochromatin-associated HP1α (17) and exerted a significant, although incomplete, effect in dispersing nuclear speckles (27). Together, these data support the model that membraneless compartments sensitive to 1,6-HD partially contribute to the higher-order chromatin organization.

Our analysis reveals that HDS-boundary clusters are enriched for genes commonly transcribed at high levels in most cell types (*SI Appendix*, Fig. S4 *B–D*). We, therefore, hypothesize that these highly transcribed genes, critically required in most cell types, have become organized in HDS-sensitive, highly accessible transcriptional boundaries in association with membraneless nuclear compartments, ensuring the robust expression of critical genes required in most human cell types. Indeed, these findings are consistent with the emerging roles of LLPS and biomolecular condensates in cellular organization and genome compartmentalization (66, 67). Through the principle of self-assembly, this model of genome organization provides an attractive model for the spontaneous assembly of DNA chains to a hierarchically organized macromolecular structure that is amenable to precise regulation. Our studies provide initial evidence for the interrelationship between transcriptional process, topological boundary formation, and spatial genome organization. Follow-up studies will be required to understand the identity and physicochemical properties of the condensates assembled at these elements that facilitate genome organization and insights into the potential functional advantages of spatial interactions, especially as these regions are highly enriched for the housekeeping genes.

## Materials and Methods

*SI Appendix* reports methods information on the antibodies used, cell culture treatments, RT-qPCR, DNA and RNA FISH, microscopy, image analysis, ChIP-seq, PRO-seq, in situ Hi-C, Hi-C data analysis and visualization, and Cistrome analysis.

## Supplementary Material

Supplementary File

Supplementary File

Supplementary File

## Data Availability

Next-gen sequencing data have been deposited in Gene Expression Omnibus (GEO) under accession no. GSE195566 (68).
