# Supplementary material for: Long-distance association of topological boundaries through nuclear condensates
Source: Proc Natl Acad Sci U S A. 2022 Aug 1;119(32):e2206216119. doi: 10.1073/pnas.2206216119 (PMC9371644; doi:10.1073/pnas.2206216119)
Supplement: Supplementary File [file pnas.2206216119.sapp.pdf]

PNAS

Supplementary Information for

Long-distance association of topological boundaries through nuclear condensates

Amir Gamliel[1,3,@], Sreejith J. Nair[1,3,4], Dario Meluzzi[1], Soohwan Oh[1,5], Nan Jiang[2], Eugin Destici[2], Michael G. Rosenfeld[1,@]

[1] Howard Hughes Medical Institute, Department of Medicine, University of California, San Diego, La Jolla, CA 92093.
[2] Department of Neurosciences, University of California, San Diego, La Jolla, CA 92093, USA
[3]These authors contributed equally to this work.

[4] Present address: Department of Oncology, Georgetown University, Washington, D.C, 20057
[5] Present address: Department of Pharmacy, Korea University College of Pharmacy, Sejong, 30019, Republic of Korea

Corresponding Author information:
Amir Gamliel, University of California, San Diego, La Jolla, CA 92093, 858-534-5858 e-mail: agamliel@health.ucsd.edu;
Michael G. Rosenfeld, University of California, San Diego, La Jolla, CA 92093, 858-534-7192 e-mail: mrosenfeld@health.ucsd.edu;

This PDF file includes:

Supplementary Methods
Figures S1 to S4
Table S1
Legends for Datasets S1 to S2
SI References

Other supplementary materials for this manuscript include the following:

Datasets S1 to S2

Supplementary Methods

Antibodies
The antibodies in this study were: anti-SON (ab121759, Abcam), anti-Rad21 (GeneTex, GTX106012), Anti-CTCF (Active Motif, 61311).

Cell Culture and Treatment
RUES1 cells were cultured in mTeSR1 (StemCell Technologies, catalog #05850), plated in Corning Matrigel hESC-qualified Matrix (Corning, catalog #354277) in a humidified incubator with 5% CO2 at 370C. For hexanediol treatment, cells were treated with 7% 1,6-Hexanediol (1,6-HD) (Sigma, Cat#240117), 2,5-Hexanediol (2,5-HD) (Sigma, Cat # H11904) for 5 minutes. The chemical was washed away with mTeSR1 and was incubated for additional time points as indicated at 37^0C before fixing with 1% formaldehyde in PBS (for ChIP-seq, Hi-C) or collecting RNA or nuclei for PRO-seq. For transcription inhibition experiments, RUES1 cells were treated with either 5μM flavopiridol hydrochloride (F3055 Sigma) or 1μM triptolide (T3652 Sigma) for 15 minutes, 1 hour, and 3 hours. Cells were then washed with PBS, cross-linked with 1% formaldehyde in PBS at room temperature for 10 min, and neutralized with 0.125M glycine. Cell pellets were stored at -80^0 C until processing for in situ Hi-C.

RT-QPCR
RNA was isolated using Trizol (Invitrogen) or RNeasy column (Qiagen), and total RNA was reverse-transcribed using SuperScript® III Reverse Transcriptase (Invitrogen). Quantitative PCRs were performed with MX3000P (Stratagene) using the VeriQuest Fast SYBR Green qPCR master mix (Affymetrix, Cat# 75690). The relative gene expression was normalized to GAPDH or beta-actin. Experiments were performed with three independent biological replicates and three technical replicates. Statistical analysis was performed using a paired two-tailed Student's t-test.

DNA FISH
DNA FISH was performed essentially as described (1). Briefly, RUES1 cells grown on gelatin-coated coverslips were treated with 2,5 hexanediol or 1,6-hexanediol (7%) for 5 minutes. The cells were then fixed with 4% paraformaldehyde in PBS for 8 min. Excess formaldehyde was quenched with 0.1M Tris-HCl (pH 7.4) for 5 min. Coverslips were washed with PBS and stored at 4^0C until used. Before hybridization, coverslips were incubated in 0.1N HCl for 5min at room temperature and washed twice with PBS. Coverslips were incubated in PBS containing 100μg/ml RNAse A for one hour at 37^0C, followed by equilibration in 50% formamide/2XSSC for 1hr. 125ng of the probe in an equal volume mixture of formamide and 2X hybridization buffer mix (4XSSC/40%Dextran Sulphate) was used per coverslip. Coverslips on glass slides were heated for 6 min on a hotplate with the temperature set at 80^0C, followed by overnight hybridization at 37ºC in a humidified dark chamber. The coverslips were then washed twice with pre-warmed buffer containing 50% formamide/2XSSC and twice with 2XSSC before being finally mounted with Vectashield antifade mounting medium with DAPI (Vector Laboratories). For ImmunoFISH (DNA), cells were incubated first with PBS containing 0.5% Triton-X-100 and 5%BSA for 15 min at room temperature. SON antibody (ab121759, Abcam) was used at a dilution of 1:100 in blocking buffer (0.1% Triton-100/5% BSA in PBS) for one hr. at 37^0C and washed three times in PBST (PBS containing 0.1% Triton X-100). They were incubated at room temperature with appropriate fluorescent conjugated secondary antibody (1:1000) dilution for 30 minutes. Cells were fixed for a second time with freshly prepared 2% paraformaldehyde for 10 min at room temperature, followed by treatment with 0.1M Tris-HCl (pH 7.4) for 5 minutes. Washed twice in PBS, and the DNA FISH protocol described above was resumed.

DNA and RNA FISH Probes
All the BAC clones for DNA FISH were from CHORI (Oakland, CA, USA). The hybridization probes for DNA FISH were generated from 1 μg BAC DNA using Nick Translation kit (Abbot Molecular), Green 496, Orange 552, or Red 650 conjugate dUTP following manufacture recommended protocol. 125ng of each labeled probe, 4μg human Cot1 DNA (Thermo Fisher Scientific), and 10μg salmon testis DNA (Sigma-Aldrich) were used per coverslip. They were co-precipitated in ethanol and were resuspended in an equal volume mixture of formamide and 2X hybridization buffer mix

(4XSSC/40%Dextran Sulphate) before hybridization reaction. BAC and Fosmid clone ID used in this study is given in ***SI Appendix* Table S1**.

Microscopy

Images were acquired using a Perkin Elmer Spinning Disk Confocal Microscope (100x Nikon Plane Apochromatic oil immersion objective, numerical aperture: 1.40). The microscope was equipped with a Piezo-Z drive and EMCCD Hamamatsu 14-bit 1Kx1K camera. Z-stack data was acquired at a step size of 150 nm. The 3D images were reviewed and processed using Volocity software (Perkin Elmer, v6.0.1). Background-subtracted image stacks were used for downstream analysis using Volocity software and custom scripts.

Image analysis

3D image stacks were initially analyzed using Volocity software. The functions “Find Object” and “Exclude Objects by Size” were combined to detect the FISH probe signals automatically. The software calculated the spatial distance between the edges of the DNA signal and SON. The data were exported to CSV files using the Volocity software and were analyzed and plotted using R.

ChIP-seq

Briefly, approximately 10^7 cells were cross-linked with 1% formaldehyde in PBS at room temperature for 10 minutes and neutralized with 0.125M glycine. After sonication, ~50μg soluble chromatin was incubated with 1-5μg of antibody at 4^0C overnight. Immunoprecipitated complexes were collected using Dynabeads A/G (Invitrogen). Subsequently, immuno-complexes were washed, DNA extracted, and purified by QIAquick Spin columns (Qiagen). For ChIP-seq, the extracted DNA was ligated to Illumina UDI adaptors followed by deep sequencing with Illumina’s HiSeq 4000 according to the manufacturer’s instructions.
Sequencing reads were inspected for quality control using FASTQC (https://www.bioinformatics.babraham.ac.uk/projects/fastqc/), and sequencing adaptors were trimmed, if necessary, using TRIMMOMATIC (http://www.usadellab.org/cms/?page=trimmomatic). Reads were aligned to hg19 with Bowtie2(2) (version 2.26) using --very-sensitive setting. Tag directories were then generated using HOMER (3) (version 4.10.3), keeping only unique aligned reads per genome position, allowing one unique read per position (-tbp 1).

Identification of ChIP-seq Peaks

ChIP-seq peaks were called using HOMER (3) findPeaks subroutine with the default settings (-style factor -o auto). The threshold was set at a false discovery rate (FDR) of 0.001 determined by peak finding using randomized tag positions in a genome with an effective size of 2×10^9 bp. Bedgraph files were generated using HOMER scripts makeUCSCfile and makeMultiWigHub.pl for visualization in the UCSC genome browser. The total number of mappable reads was normalized to 10^7 for each experiment presented. Heatmaps were generated using deeptools2 (4) (version 3.4.3).

Global run-on sequencing (GRO-seq)

GRO-Seq experiments were performed as previously reported (5). Briefly, ~10 million RUES1 cells were washed three times with cold PBS and then sequentially swelled in swelling buffer (10mM Tris-HCl pH7.5, 2mM $MgCl_2$, 3mM $CaCl_2$) for 5 minutes on ice, then lysed in lysis buffer (swelling buffer plus 0.5% NP-40 and 10% glycerol). The resultant nuclei were washed one more time with 10mL lysis buffer and finally resuspended in 100uL of freezing buffer (50mM Tris-Cl pH8.3, 40% glycerol, 5mM $MgCl_2$, 0.1mM EDTA). For the run-on assay, re-suspended nuclei were mixed with an equal volume of reaction buffer (10mM Tris-Cl pH 8.0, 5mM $MgCl_2$, 1mM DTT, 300mM KCl, 20 units of SUPERase-IN, 1% sarkosyl, 500μM ATP, GTP, and Br-UTP, 2μM CTP) and incubated for 5 min at 30°C. The resultant nuclear-run-on RNA (NRO-RNA) was then extracted with TRIzol LS reagent (Invitrogen) following the manufacturer’s instructions. NRO-RNA was fragmented to ~300-500nt by alkaline base hydrolysis on ice and followed by treatment with DNase I and Antarctic phosphatase. These fragmented Br-UTP labeled nascent RNA was then immunoprecipitated with anti-BrdU agarose beads (sc-32323AC, Santa Cruz Biotechnology) in binding buffer (0.5XSSPE, 1mM EDTA, 0.05% tween) for three hours at 4°C with rotation. Purified RNA was treated with PNK

before being used for cDNA synthesis using NEBNext® Multiplex Small RNA Library Prep Set for Illumina® Kit (NEB). Obtained cDNA template was amplified by PCR using the LongAmp® Taq 2X Master Mix (NEB) for deep sequencing.

Precision Run-On Sequencing (PRO-seq):
PRO-seq experiments were performed as previously described (6) with the following modifications. For nuclei isolation, ~10 million RUES1 cells were incubated with swelling buffer (10 mM Tris-HCl pH7.5, 2 mM $MgCl_2$, 3 mM $CaCl_2$) for 5 minutes on ice and then incubated with lysis buffer (swelling buffer with 0.5% NP-40 and 10% glycerol) for 5 minutes on ice, before being re-suspended in 100µl of freezing buffer (50 mM Tris-Cl pH8.0, 40% glycerol, 5 mM $MgCl_2$, 0.1 mM EDTA). For the run-on assay, an equal volume of reaction buffer (10 mM Tris-HCl pH 8.0, 5 mM $MgCl_2$, 300 mM KCl, 1 mM DTT, 20 units of SuperaseIn, 1% sarkosyl, 500 µM ATP, GTP, bio-UTP, and bio-CTP) was added into each sample before incubation at 30°C for 5 min. The nuclear run-on RNA was then extracted with TRIzol LS reagent (Invitrogen) and subjected to hydrolysis, buffer exchange, and purification by streptavidin beads (Thermo Fisher). Purified RNA was treated with PNK before being used for cDNA synthesis using NEBNext® Multiplex Small RNA Library Prep Set for Illumina® Kit (NEB). Obtained cDNA templates were amplified by PCR using the LongAmp® Taq 2X Master Mix (NEB) for deep sequencing.
Sequencing reads were inspected for quality control using FASTQC (https://www.bioinformatics.babraham.ac.uk/projects/fastqc/), and sequencing adaptors were trimmed, if necessary, using TRIMMOMATIC (http://www.usadellab.org/cms/?page=trimmomatic). Reads were aligned to hg19 with Bowtie2 (2) (version 2.26) using --very-sensitive setting. Tag directories were then generated using HOMER (3) (version 4.10.3), keeping only unique aligned reads per genome position but allowing up to 3 unique reads per position (-tbp 3). For read counting, the HOMER script analyzeRepeats.pl was used to estimate the raw counts per gene and also to compute the pausing ratio. The aligned reads were counted over the RefSeq gene bodies (after excluding a TSS 400bp-proximal region downstream of TSS up to 13Kb of the gene body). EdgeR (http://www.bioconductor.org/) was used to compute the significance of the differential gene expression (FC≥1.5, FDR≤0.05). Additionally, a read density threshold (i.e., GRO-seq normalized read counts/kb) was used to exclude lowly expressed genes.
Bedgraph files were generated using HOMER scripts makeUCSCfile and makeMultiWigHub.pl for visualization in the UCSC genome browser. Heatmaps were generated using deeptools2(4) (version 3.4.3).

In-situ Hi-C
In situ Hi-C was performed as described (7) using MboI restriction enzyme for hexanediol Hi-C or DpnII for flavopiridol and triptolide Hi-C. Hi-C DNA was purified on Dynabeads MyOne Streptavidin T1 beads (Thermo Fisher), and sequencing libraries were prepared using the KAPA HTP/LTP Library Preparation Kits. Hi-C libraries were sequenced by paired-end sequencing on an Illumina HiSeq 4000 system, using 75 bp per read in each pair, or NovaSeq, using 100 bp paired-end reads. Data were analyzed with juicertools (8) (version 1.6.2) for visualization purposes or as described below.

Hi-C data analysis
Construction of contact maps
Read pairs obtained by sequencing Hi-C libraries were mapped to the human reference genome assembly hg19 using bowtie2 (2) version 2.3.0 with the following parameters: --end-to-end --very-sensitive -L 30 --score-min L,-0.6,-0.2 --mp 6,2. For each pair, each 100-base read was mapped independently. Reads aligning to multiple genomic locations were discarded. Reads that could not be aligned to any genomic location were further processed. The 8-base sequence (GATCGATC), expected for the junction resulting from ligation of the blunt ends of two MboI restriction fragments, was searched within the read sequence, allowing for a single base mismatch. If the searched junction sequence was found at one or two locations, the read sequence was split at the midpoint of each junction, and the resulting sub-sequences were mapped independently to the reference genome. If the junction sequence was not found, the read sequence was processed in the iterative mapping procedure described by Imakaev et al. (74). Sub-sequences starting from each end of the

read were iteratively extracted and independently mapped to the reference genome. The initial length of each sub-sequence was 16 bases and was incremented by five bases until the sub-sequence did not align to multiple genomic locations. All read sequences and sub-sequences that successfully aligned to unique genomic locations were grouped by reading pair of origin. Groups greater than one were analyzed to infer pairs of reading sequences or sub-sequences that likely resulted from cross-linked restriction fragments. For each inferred aligned (sub-)sequence pair, the two alignment coordinates were used to filter out duplicated pairs, likely resulting from PCR amplification, and spurious intra-chromosomal contacts, likely resulting from dangling ends and self-ligation products, as described in Jin F et al. (9). Specifically, sequence pairs aligned to genomic locations closer than 1000 bp were classified as “same strand” and “outward” pairs and hence discarded. The remaining aligned sequence pairs were used to generate raw contact maps, i.e., symmetric matrices of raw contact counts for all possible pairs of genomic bins (10), with one matrix per chromosome and either 40 kb or 250 kb per bin. The contact maps were inspected to identify rows and columns that contained too many zero entries for all of the analyzed Hi-C libraries. The minimum acceptable number of non-zero entries was arbitrarily set to 25% of the median number of non-zero entries in each row. The identified rows and columns were omitted from subsequent analysis steps. To remove experimental biases in the matrices (11), each raw contact map was processed using an iterative matrix-balancing procedure that yields a matrix with approximately constant row and column sums (12) (10).

Reproducibility of Hi-C data

The reproducibility of the Hi-C experiments was assessed through a modified calculation of the “stratum-adjusted correlation coefficient” (SCC) (13) for each pair of raw contact maps. The SCC is a weighted average of Pearson correlation coefficients, each calculated using contact counts for a given genomic distance after averaging the contact map with a 2D mean filter. Smoothing the contact maps reduces local noise, whereas stratifying the correlation coefficient by genomic length prevents the short-distance contact counts from dominating the final result. However, the smoothing performed in the original calculation of the SCC does not account for the decreasing trend of contact counts with genomic distance, leading to excessive smoothing at short distances and insufficient smoothing at large distances. The raw contact maps were not pre-processed with a 2D mean filter to address this issue. Instead, the correlation coefficient was calculated for ten contiguous and increasing ranges of genomic distances. The extent of each range was calculated to achieve an approximately constant contact sum within each range, assuming a power-law dependence of contact counts on genomic distance with exponent -1. The smallest range of distances extended from 200 kb to 280 kb, and the largest range extended from 4.04 Mb to 5.52 Mb. The contact counts were averaged within each range along the rows or columns of the contact maps. The correlation coefficient was calculated separately for each of the two averaging directions. The correlation coefficients thus obtained for all ranges of genomic distances and all chromosomes were averaged as previously described (13). The SCC was calculated for each pair of Hi-C libraries and subtracted from 1 to obtain a measure of the distance between the Hi-C libraries in each pair. The resulting distances were used to perform hierarchical clustering with function scipy.cluster.hierarchy.complete from package SciPy (14) version 1.7.1.

Calculation of insulation score

To calculate the insulation score at a given genomic bin from a given contact map, the elements of the matrix containing the contact map were summed within a square window (A in figure 1c), with a corner touching the main diagonal of the matrix at the location of the given genomic bin. The resulting sum, known as the insulation score (15), was then divided by the sums of the elements within the two triangular windows (B and C in Figure 1c) immediately preceding and following the square window along the main diagonal of the matrix. This calculation is equivalent to ignoring the elements of the main diagonal in the calculation of the score used by Alekseyenko et al. (16) and then taking the reciprocal of that score. The resulting ratio quantifies the frequency of chromatin contacts between the two regions on each side of a given genomic bin (Region 1 and Region 2 in Figure 1c) relative to the frequency of contacts within those regions. This relative contact frequency is less sensitive to sample-dependent variations in read counts over broad genomic regions that encompass multiple chromatin domains and is a robust measurement for comparing domain boundary strength across experimental conditions.

Identification of domain boundaries

To identify the locations of contact domain boundaries on a given chromosome, the unbiased contact map for that chromosome, at a resolution of 40 kb per genomic bin, was used as input for calculating the insulation score. The estimated first derivative of the insulation score signal at each bin was obtained through a linear fit over a sliding window of 9 genomic bins. The zero-crossings and signs of the estimated first derivative were used to identify bins corresponding to local maxima and minima of the insulation score. As needed for the next step, local maxima and minima were used to calculate the median peak-to-peak insulation score on each chromosome. To select sufficiently strong domain boundaries, the insulation score at each local minimum was subtracted from the insulation score at each of the two local maxima adjacent to that local minimum, the larger of the two differences was identified, and the local minimum was retained if such difference exceeded 20% of the median peak-to-peak interaction score. Pairs of adjacent genomic bins containing a local minimum of insulation score were iteratively replaced with the bin with the smallest insulation score until all such pairs were eliminated. The remaining bins containing local insulation score minima were taken as the genomic locations of contact domain boundaries on the given chromosome.

Determination of significant changes in domain boundaries

A master list of domain boundaries was generated using the contact maps from cells treated with 2,5-HD. These contact maps were obtained by combining ~173M aligned read pairs from two biological replicates of Hi-C experiments. The interaction score at each identified boundary was tested for significant changes in each comparison between the 2,5-HD treatment and a time point (5 min, 30 min, 3 hours) after 1,6-HD treatment. To perform the test at a given boundary, a generalized linear model (GLM) with quasibinomial response variable and logit link function was fit to the raw contact counts in the numerator and denominator of the interaction score formula (Figure 1c). The experimental condition (2,5-HD treatment or time point after 1,6-HD treatment) was used as the explanatory variable, with two replicates per condition. The fit was carried out using the function glm in the R environment (17) version 4.0.2. The p-value of the regression coefficient corresponding to each time point after 1,6-HD treatment was used to assess the statistical significance of the change in interaction score at the given domain boundary for the given time point. The p-values obtained for a given time point at all domain boundaries were adjusted for multiple testing using the method of Benjamini and Hochberg (18), as implemented by the function p.adjust in the R environment (17). Domain boundaries with adjusted p-values less than a false discovery rate of 1% were deemed to be significantly changed at the given time point after 1,6-HD treatment.

Calculation of A/B compartment profiles

To identify A/B-compartments in each chromosome, the matrix of unbiased contact counts binned at 500-kb or 40-kb resolution for a given chromosome was first smoothed using a Gaussian filter with a standard deviation of one bin. Each element in the smoothed matrix was converted to a z-score by calculating the local mean and standard deviation within a 20-Mb segment centered on that element and parallel to the main diagonal of the matrix. The main and first adjacent diagonals of the resulting z-score matrix were set to zero to avoid excessive influence from the large values in those diagonals. A matrix of Pearson correlation coefficients was then computed using all possible pairs of rows and columns in the z-score matrix. The first eigenvector of the covariance matrix of the Pearson correlation matrix was calculated using SciPy (14) function scipy.sparse.linalg.eigsh to obtain the magnitude of the desired A/B-compartment profile for the given chromosome. By convention (19), positive values of the A/B-compartment profile correspond to A-compartments, which are believed to be rich in gene promoters and thus have high GC content. Therefore, to obtain the final A/B-compartment profile for the control sample (2,5-HD treatment) at 500-kb resolution, the sign of the eigenvector was corrected as needed to produce a positive covariance with a profile of GC density collected from the hg19 reference sequence of the given chromosome. To obtain the final A/B-compartment profile for all samples at the 40-kb resolution, the sign of the eigenvector was corrected to produce a positive covariance with the A/B-compartment profile obtained for the control sample at 500-kb resolution.

Comparison of A/B compartment profiles

To identify statistically significant changes in A/B-compartment profiles, a linear model was fit to those profiles at each genomic bin by using function lmFit from package limma (20) version 3.44.3 in the R environment (17) version 4.0.2. Each of the 5726 rows in the response variable matrix

passed to function lmFit corresponded to a genomic bin. At the same time, each of the eight columns contained the A/B-compartment profile at 500-kb resolution from one Hi-C library corresponding to one of two replicates per experimental condition. The limma function eBayes was then used with default parameters to perform empirical Bayes moderation of standard errors (21) and to identify genomic bins having a significant change in A/B compartment profile when comparing each time point after 1,6-HD treatment to the 2,5-HD treatment condition. To adjust the resulting p-values for multiple testing, the limma function top table was used with default parameters, thus applying the method of Benjamini and Hochberg (18).

Inter-chromosomal interactions

Each chromosome was subdivided into contiguous 4-Mb bins to investigate inter-chromosomal interactions between A-compartments. Each such bin was labeled as A- or B-compartment according to whether the average of the A/B-compartment profile at a 40-kb resolution within that bin was greater than or less than zero, respectively. Each genomic bin was further labeled with the number of HDS boundaries found. To avoid excessively high contact counts arising near the centromeric regions, those regions were extended by 1Mb in each direction. Any 4-Mb genomic bins overlapping the extended regions were omitted from subsequent analysis steps. The coordinates of the centromeric regions were extracted from the file https://hgdownload.cse.ucsc.edu/goldenPath/hg19/database/cytoBand.txt.gz, provided by the UCSC Genome Browser (22). The remaining 4-Mb genomic bins from all chromosomes, except chrY, were used to build a genome-wide raw contact map for each Hi-C library by counting pairs of sequences aligned to each possible pair of such bins. The contact maps obtained from the two replicate Hi-C libraries for each experimental condition were combined by matrix addition. The resulting raw contact map was subjected to the same matrix balancing procedure used for the intra-chromosomal contact maps, thus obtaining contact maps with constant row and column sums. Each unbiased contact map was rescaled to equalize such sums across experimental conditions to achieve a normalized contact count of 160 per pair of 4-Mb genomic bins. Specifically, each contact map was divided by its sum and multiplied by ten times the square of the total covered genomic length in Mb. A heat-map representation of the normalized contact map for the control condition (2,5-HD treatment) was generated using Python package matplotlib (23) version 3.4.2. All possible pairs of 4-Mb genomic bins labeled as belonging to the A-compartment were selected, and the distribution of normalized contact counts corresponding to those pairs of bins was visualized using one box plot for each experimental condition and each range of several HDS boundaries present within each bin (Figure 4e, f). The box plots were generated using the R package ggplot2 (24) versions 3.3.3 and 3.3.5. The distributions of normalized contact counts were compared using the Wilcoxon rank-sum test through the R function wilcox.test.

Hi-C visualization

40kb bins from all chromosomes, except chrY, were used to build a genome-wide raw contact map for each Hi-C library. The contact maps obtained from the two replicate Hi-C libraries for each experimental condition were combined by matrix addition. The resulting raw contact map was subjected to matrix balancing. Heat-map representations of the normalized contact map were generated using Python package matplotlib(23) version 3.4.2. Matrices were also visualized using Juicebox (25) version 1.9.8 or Fan-C plot(26) version 0.9.0. Significant interactions were called using fit-Hi-C(27) (version 1.2.0) and plotted on the WashU epigenome browser (https://epigenomegateway.wustl.edu/).

Cistrome analysis

For cistrome analysis, we utilized Cistrome DB, also known as Cistrome Data Browser, an interactive database that enables visualization of public ChIP-seq, DNase-seq, and ATAC-seq data (28, 29). Briefly, 949 40kb HDS boundaries or 4568 40kb non-HDS boundaries (***SI Appendix* Dataset S2, qval<0.05**) were converted to hg38 using the UCSC liftover tool (http://genome.ucsc.edu/cgi-bin/hgLiftOver) and input into the DB toolkit section (http://dbtoolkit.cistrome.org/), using the options to find factors that have a significant binding overlap of transcription factor and chromatin regulators, with the boundary (peak) set, using the top 10k peaks according to peak enrichments. GIGGLE software (30) was used to search the Cistrome database and return samples that contain peak sites on the interval. The resulting GIGGLE score is essentially the ranking of the significance and enrichment of the factors in the

database to the queried regions, where higher GIGGLE scores indicate more enrichment. Each dot on the resulting plot represents a ChIP-seq sample with its corresponding GIGGLE score.

Data availability

Next-gen sequencing data sets generated from this study can be accessed at GEO using accession ID GSE195566.

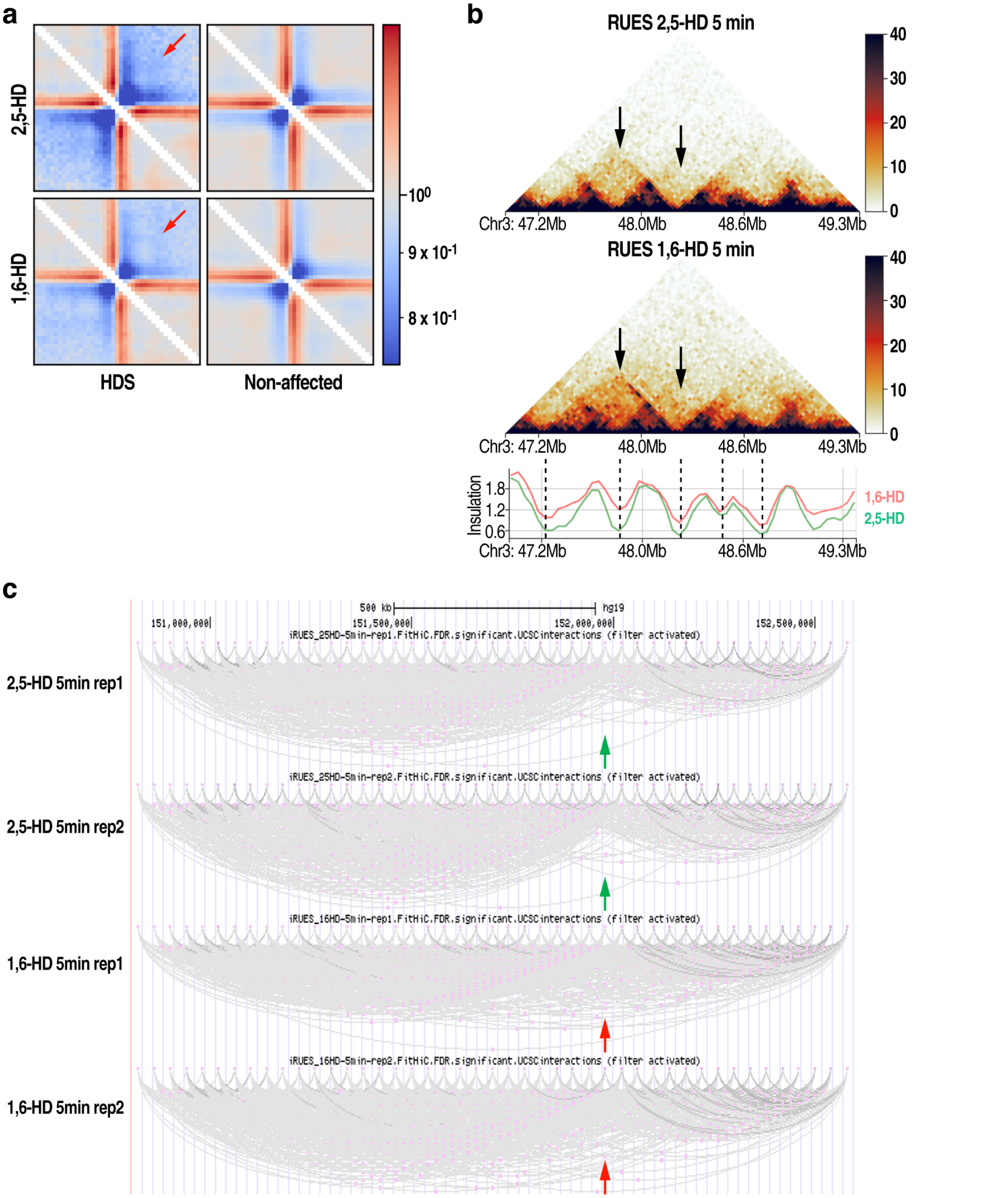

Figure S1. a. Hi-C aggregate interaction plots centered on either HDS-boundaries or unaffected boundaries in control (2,5-HD) or 1,6-HD treated RUES cells. Insulation is greater in control vs. 16HD treatment (darker blue indicates greater insulation, red arrow) only on HDS boundaries, not on unaffected boundaries. b. Triangular Hi-C matrix plot showing increased interactions across boundaries following 1,6-HD treatment (arrows). Below, insulation scores are plotted for 1,6-HD (red) and control (2,5-HD, green). c. Example region plotting significant interactions determined by diffHiC across an HDS boundary showing few cross-boundary interactions in the control 2,5-HD condition (green arrows) vs. the 1,6-HD treatment condition (red arrows).

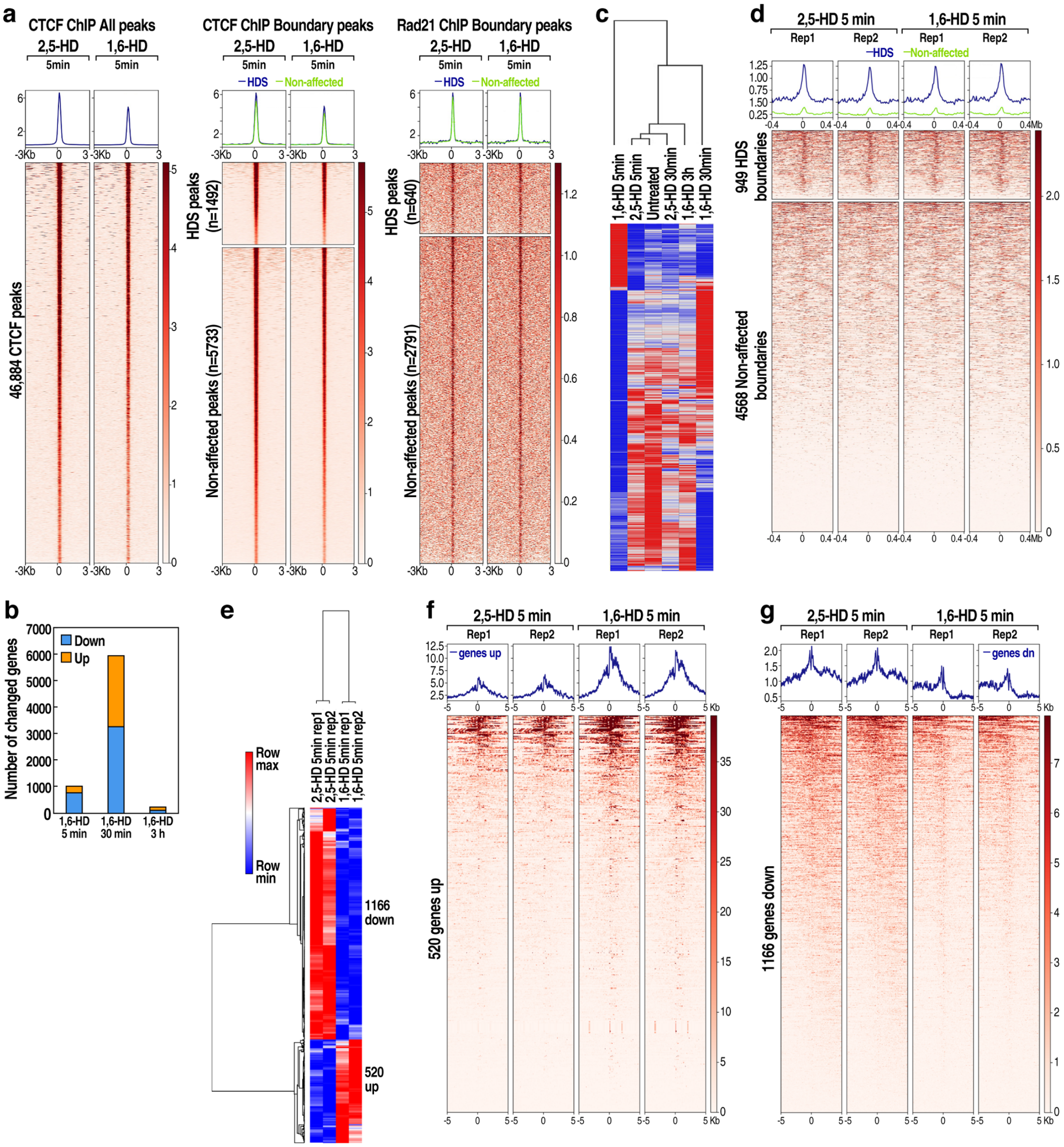

Figure S2. a. CTCF ChIP-seq heatmaps of all CTCF ChIP-seq peaks in 2,5-HD control or 1,6-HD conditions (left), heatmaps of ChIP-seq peaks overlapping either HDS or unaffected boundaries (CTCF, middle), (Rad21, right). b. The number of significantly up and down-regulated genes after treatment with 1,6-HD at the different time points. c. Clustering of gene significantly changed after 5 minutes of 1,6-HD treatment (high expression -red, low expression – blue). d. heatmap of PRO-seq tags centered on boundaries in replicates after 5 minutes of 1,6-HD treatment vs. control (2,5-HD), on HDS-boundaries (top) and unaffected boundaries (bottom). e. Heatmap of expression values from PRO-seq analysis showing significantly downregulated genes after 5 minutes of 1,6-HD treatment (top) vs. up-regulated genes (bottom). f. Heatmaps of PRO-seq tags of significantly up-regulated genes centered on their transcription start site (TSS). g. Heatmaps of PRO-seq tags of significantly down-regulated genes centered on their transcription start site (TSS).

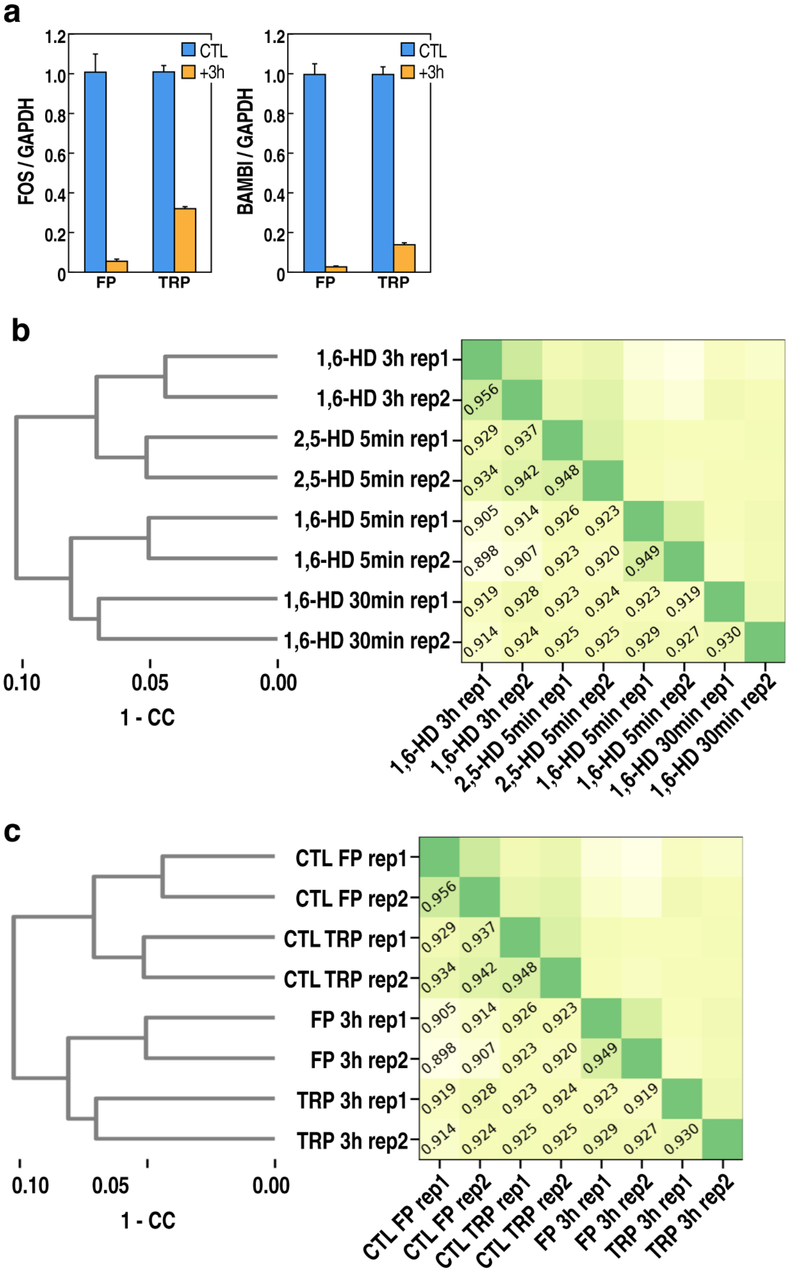

Figure S3. a. qRT-PCR mRNA expression levels for FOS (left), and BAMBI (right) normalized to GAPDH RNA, showing inhibition of transcription following 3-hour flavopiridol and triptolide treatment. b. Correlation coefficient clustering of Hi-C matrices shows high concordance between replicated treated with 1,6-HD and 2,5-HD. c. Correlation coefficient clustering of Hi-C matrices showing high concordance between replicated treated with flavopiridol and triptolide.

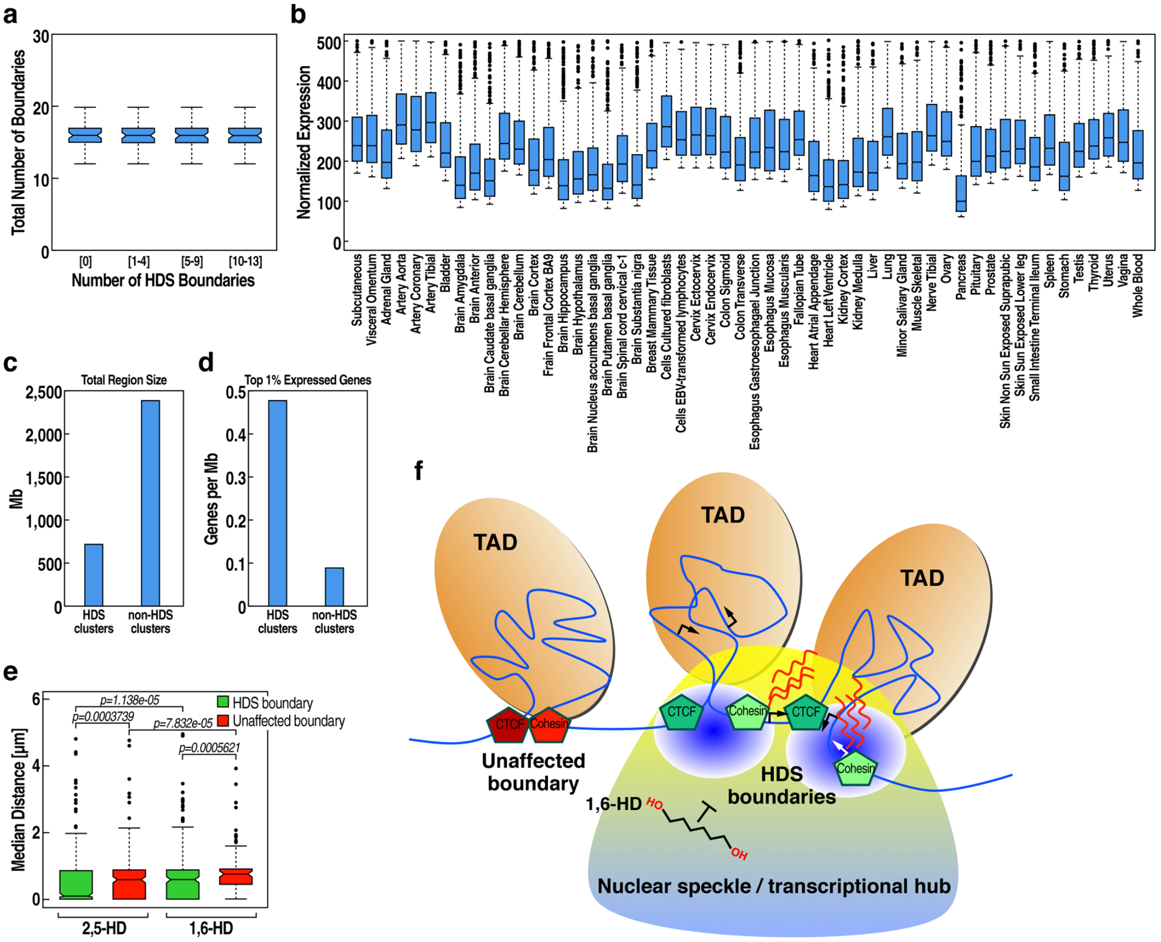

Figure S4. a. Distribution of the number of total boundaries in each 4Mb region grouped by the number of HDS boundaries per region, showing an equivalent number of boundaries in each group. **b**. RNA-seq expression levels in tags per million (TPM) of the top 1% of common highly expressed transcripts (546 genes) in all 54 tissues in the human GTEx database. **c**. Total genomic region size of all HDS and non-HDS clusters. **d**. Enrichment of the top 1% common genes over HDS clusters vs. non-HDS clusters. The number of genes was normalized to the region size as shown in panel **c**. **e**. Quantitation of immune-DNA FISH images showing that HDS boundary clusters (green) are significantly closer to nuclear speckle marker (SON) than unaffected boundaries (red) **f.** Model depicting boundaries demarcated by CTCF and cohesin (SMC), with HDS boundaries showing increased transcription (red wavy lines) in proximity to nuclear speckles (yellow area).

Table S1. List of BAC clones used for DNA-FISH analysis

	**BAC clone ID**
**Experiment I**	
HDS-boundary	RP11-581C12
Non-HDS-boundary	RP11-638O18
**Experiment II**	
HDS-boundary	RP11-664L8
Non-HDS-boundary	RP11-590C10

Dataset S1 (separate file). Summary of Hi-C experiments

Dataset S2 (separate file). List of TAD boundaries in RUES1 cells treated with 1,6-hexanediol

SI References:

1. S. J. Nair *et al.*, Phase separation of ligand-activated enhancers licenses cooperative chromosomal enhancer assembly. *Nat Struct Mol Biol* **26**, 193-203 (2019).
2. B. Langmead, S. L. Salzberg, Fast gapped-read alignment with Bowtie 2. *Nature Methods* **9**, 357-359 (2012).
3. S. Heinz *et al.*, Simple combinations of lineage-determining transcription factors prime cis-regulatory elements required for macrophage and B cell identities. *Mol Cell* **38**, 576-589 (2010).
4. F. Ramirez *et al.*, deepTools2: a next generation web server for deep-sequencing data analysis. *Nucleic Acids Res* **44**, W160-165 (2016).
5. S. Oh *et al.*, Enhancer release and retargeting activates disease-susceptibility genes. *Nature* 10.1038/s41586-021-03577-1 (2021).
6. D. B. Mahat *et al.*, Base-pair-resolution genome-wide mapping of active RNA polymerases using precision nuclear run-on (PRO-seq). *Nat Protoc* **11**, 1455-1476 (2016).
7. S. S. Rao *et al.*, A 3D map of the human genome at kilobase resolution reveals principles of chromatin looping. *Cell* **159**, 1665-1680 (2014).
8. N. C. Durand *et al.*, Juicer Provides a One-Click System for Analyzing Loop-Resolution Hi-C Experiments. *Cell Syst* **3**, 95-98 (2016).
9. F. Jin *et al.*, A high-resolution map of the three-dimensional chromatin interactome in human cells. *Nature* **503**, 290-294 (2013).
10. B. R. Lajoie, J. Dekker, N. Kaplan, The Hitchhiker's guide to Hi-C analysis: Practical guidelines. *Methods* **72**, 65-75 (2015).
11. E. Yaffe, A. Tanay, Probabilistic modeling of Hi-C contact maps eliminates systematic biases to characterize global chromosomal architecture. *Nat Genet* **43**, 1059-1065 (2011).
12. M. Imakaev *et al.*, Iterative correction of Hi-C data reveals hallmarks of chromosome organization. *Nat Meth* **9**, 999-1003 (2012).
13. T. Yang *et al.*, HiCRep: assessing the reproducibility of Hi-C data using a stratum-adjusted correlation coefficient. *Genome Research* **27**, 1939-1949 (2017).
14. P. Virtanen *et al.*, SciPy 1.0: fundamental algorithms for scientific computing in Python. *Nature Methods* **17**, 261-272 (2020).
15. E. Crane *et al.*, Condensin-driven remodelling of X chromosome topology during dosage compensation. *Nature* **523**, 240-244 (2015).
16. A. A. Alekseyenko *et al.*, The oncogenic BRD4-NUT chromatin regulator drives aberrant transcription within large topological domains. *Genes & Development* **29**, 1507-1523 (2015).

17. R Core Team (2021) R: A Language and Environment for Statistical Computing. (R Foundation for Statistical Computing, Vienna, Austria).
18. Y. Benjamini, Y. Hochberg, Controlling the False Discovery Rate: A Practical and Powerful Approach to Multiple Testing. *Journal of the Royal Statistical Society: Series B (Methodological)* **57**, 289-300 (1995).
19. E. Lieberman-Aiden *et al.*, Comprehensive Mapping of Long-Range Interactions Reveals Folding Principles of the Human Genome. *Science* **326**, 289-293 (2009).
20. M. E. Ritchie *et al.*, limma powers differential expression analyses for RNA-sequencing and microarray studies. *Nucleic Acids Res* **43**, e47-e47 (2015).
21. G. K. Smyth, Linear Models and Empirical Bayes Methods for Assessing Differential Expression in Microarray Experiments. *Statistical Applications in Genetics and Molecular Biology* **3** (2004).
22. W. J. Kent *et al.*, The Human Genome Browser at UCSC. *Genome Research* **12**, 996-1006 (2002).
23. J. D. Hunter, Matplotlib: A 2D Graphics Environment. *Computing in Science & Engineering* **9**, 90-95 (2007).
24. H. Wickham, *ggplot2: Elegant Graphics for Data Analysis* (Springer-Verlag New York, 2016).
25. N. C. Durand *et al.*, Juicebox Provides a Visualization System for Hi-C Contact Maps with Unlimited Zoom. *Cell Syst* **3**, 99-101 (2016).
26. K. Kruse, C. B. Hug, J. M. Vaquerizas, FAN-C: a feature-rich framework for the analysis and visualisation of chromosome conformation capture data. *Genome Biol* **21**, 303 (2020).
27. F. Ay, T. L. Bailey, W. S. Noble, Statistical confidence estimation for Hi-C data reveals regulatory chromatin contacts. *Genome Res* **24**, 999-1011 (2014).
28. R. Zheng *et al.*, Cistrome Data Browser: expanded datasets and new tools for gene regulatory analysis. *Nucleic Acids Res* **47**, D729-D735 (2019).
29. S. Mei *et al.*, Cistrome Data Browser: a data portal for ChIP-Seq and chromatin accessibility data in human and mouse. *Nucleic Acids Res* **45**, D658-D662 (2017).
30. R. M. Layer *et al.*, GIGGLE: a search engine for large-scale integrated genome analysis. *Nat Methods* **15**, 123-126 (2018).
